# Delta‐like protein 1 in the pituitary‐adipose axis in the adult male mouse

**DOI:** 10.1111/jne.12507

**Published:** 2017-08-28

**Authors:** A. R. Bello, R. A. Puertas‐Avendaño, M. J. González‐Gómez, M. González‐Gómez, J. Laborda, C. Damas, M. Ruiz‐Hidalgo, C. Diaz

**Affiliations:** ^1^ Cell Biology Section School of Sciences/Institute for Tropical Diseases and Public Health University of La Laguna Tenerife Spain; ^2^ Department of Inorganic and Organic Chemistry and Biochemistry School of Medicine/Regional Centre for Biomedical Research Biomedicine Unit Spanish National Research Council/University of Castilla‐La Mancha Albacete Spain; ^3^ Department of Basic Medical Sciences School of Medicine University of La Laguna Tenerife Spain; ^4^ Department of Psychobiology School of Psychology University of La Laguna Tenerife Spain; ^5^ Department of Medical Sciences School of Medicine/Institute for Research in Neurological Disabilities University of Castilla‐La Mancha Albacete Spain

**Keywords:** anterior pituitary, hormone receptors, leptin, leptin receptor

## Abstract

With the aim of studying delta‐like protein 1 (DLK1) with respect to the relationship between adipocyte leptin and adenohypophyseal hormones, we carried out an immunohistochemical study analysing the presence of receptors for these hormones in the pituitary and adipose cells of male wild‐type (WT) mice (*Dlk1* ^+/+^) compared to knockout (KO) mice (*Dlk1* ^−/−^). The mRNA expression of these molecules was also determined using the reverse transcriptase‐polymerase chain reaction. The results obtained showed that, in WT adipose cells, all of the adenohypophyseal hormone receptors were present, with a higher mRNA expression for growth hormone (GH) receptor and thyroid‐stimulating hormone (TSH) receptor. Of the total cells in the anterior pituitary lobe, 17.09±0.9% were leptin receptor (LEPR) immunoreactive (‐IR), mainly in GH‐IR and prolactin (PRL)‐IR cells (41.5±3.8%; 13.5±1.7%, respectively). In *Dlk1* ^−/−^ mice, adipocyte cells showed a significant increase in the TSH receptor mRNA expression level. Moreover, the percentage of LEPR‐IR GH cells showed a statistically significant increase compared to controls, from 41.5±3.8% to 53.1±4.0%. By contrast, only 3.0±0.6% of LEP‐IR anterior pituitary cells were detected in Dlk1 KO mice, as opposed to 6.8±1.1% observed in WT mice. The results suggest that relationships exist between adipocytes and pituitary GH, PRL and TSH cells, in addition to an influence with respect to the synthesis and release of pituitary leptin, particularly in PRL cells.

## 1 INTRODUCTION

At present, white adipose tissue is known to be an active endocrine organ producing hormones such as leptin (LEP), proinflammatory and anti‐inflammatory cytokines, and numerous other regulatory factors.^1^, ^2^ In 1994, adipose tissue was shown to produce the hormone LEP, with the *Ob* gene being discovered in mice for the first time.^3^ Furthermore, since the 1980s, evidence has accumulated supporting the presence of receptors for hormones and peptides released by the hypothalamus and pituitary gland in mature fat cells, at least *in culture*.^4^ Such findings have suggested the existence of a hypothalamic‐pituitary‐adipose axis.^5^ Subsequently, the term “adipotrophins” was introduced to describe pituitary and hypothalamic hormones that directly target adipocytes. Although follicle‐stimulating hormone (FSH) and luteinsing hormone (LH) receptors were not detected in experiments conducted in cultured adipocytes, more recently, gonadotrophin receptors have been described in chicken adipose tissue.^6^

In the pituitary gland, LEP exerts a direct or indirect effect on the secretion and regulation of the adenohypophyseal hormones gowthn hormone (GH), FSH/LH, adrenocorticotrophic hormone (ACTH), thyroid‐stimulating hormone (TSH) and prolactin (PRL).^7^, ^8^, ^9^, ^10^, ^11^, ^12^ Numerous studies show that LEP regulates the four main hypothalamic‐pituitary peripheral axes (adrenal, thyroid, gonadal and growth hormone) at different levels.^13^

Direct LEP action on the pituitary is exerted by binding to its receptor (LEPR) in endocrine cells of the anterior lobe. Thus, homozygote *LepR* mutant mice displayed lowered TSH and GH secretion.^14^ Moreover, the selective deletion of LEPR expression in hypophyseal GH cells in murine models gave rise to a reduction in both somatotroph cells and serum GH levels, whereas abdominal fat increased in adult mice.^9^ In addition, the loss of somatotroph leptin resulted in lower prolactin levels in serum of 6‐month‐old female mice.^12^ The presence of the long form of the LEPR in hypophyseal cells of the anterior lobe has been demonstrated in the rat and mouse,^15^, ^16^ although the distribution between the different cell types varies among species. In rats, 97% of GH‐producing cells express the leptin receptor, whereas less than 1% of the other cell types do so.^16^ Moreover, using dispersed cells in culture, high percentages of adenohypophyseal cells expressing LEPR have been described in rats and mice.^9^, ^17^ By contrast, in sheep, approximately 70% of somatotroph cells, as well as 30% of gonadotroph and corticotroph cells, express this receptor.^18^

In recent years, numerous studies have shown that LEP is also produced by pituitary cells,^15^, ^16^, ^19^, ^20^ suggesting a paracrine or autocrine role for this hormone in the pituitary gland. Although there is no agreement to date regarding the cell types that produce LEP,^15^, ^19^, ^21^, ^22^, ^23^ most studies concur that GH cells are the most important LEP‐producing cells in the pituitary gland. This is in agreement with the evidence indicating that LEP is important for GH secretion, as reported in several studies.^24^, ^25^ Recently, Odle et al^11^ established different roles for adipocyte and pituitary LEP in the GH axis. Their data suggest that pituitary LEP regulates the development and maintenance of somatotroph cells, whereas adipocyte LEP acts on the secretion of GH. It was also shown that adipocytes are the only source of circulating LEP.

Regarding the regulation of adipocyte LEP production, delta‐like protein 1 (DLK1) is a transmembrane protein with regulatory effects on adipocyte differentiation.^26^, ^27^, ^28^ *Dlk1* mRNA is also widely expressed during embryonic development, although, in the adult, *Dlk1* mRNA expression is limited to some endocrine glands and subsets of neurones in the brain, including the hypothalamus.^29^, ^30^, ^31^, ^32^ In the pituitary gland of the 129/Svj wild‐type (WT) mouse, we previously demonstrated that DLK1 is expressed in all types of cells, particularly in somatotroph cells.^33^, ^34^ Furthermore, we used a *Dlk1* knockout (KO) mouse of the same strain that displays a smaller pre‐ and postnatal size but increased white fat mass in the adult.^35^, ^36^ The results obtained showed increased serum leptin in these KO mice, as well as a slight increase in GH levels, despite having a smaller number of GH‐producing cells.^33^ Based on these data, the present study aimed to investigate in situ (ie, maintaining the natural context of the cells), the influence of DLK1 protein on the relationship between adipocyte and adenohypophyseal cells, using male WT and *Dlk1* ^*−*/*−*^ KO mice of the strain 129/SvJ.

## 2 MATERIALS AND METHODS

### 2.1 Animals

Eighteen adult male mice (129⁄SvJ) approximately 4 months old were used: nine WT mice (*Dlk1* ^+/+^) and nine *Dlk1* KO mice (*Dlk1* ^−/−^). All animals were supplied by the Animal House Facility of the University of Castilla‐La Mancha (Spain). *Dlk1*‐deficient mice were generated as described previously.^36^ The experimental procedures were approved by the Ethics Committee for Experimental Animal Welfare of the University of Castilla‐La Mancha and conformed with Spanish (Royal Decree 1201/2005; Law 32/2007) and European Union (Directive 2010/63/UE) regulations for the use and care of animals in research.

### 2.2 Antisera

Antibodies are listed in the Supporting information (Table S1). A polyclonal antiserum (#1125) against DLK1 raised in rabbit was used,^37^ along with polyclonal goat and rabbit anti‐DLK1 antibodies purchased from Santa Cruz Biotechnology Inc. (Heidelberg, Germany) (for details, see the Supporting information, Table S1). Polyclonal antisera for identifying the pituitary hormones ACTH, PRL, FSH and TSH were produced in rabbits by Dr G. Tramu (University of Bordeaux 1, Bordeaux, France). The specificity of these antisera was evaluated by means of an absorption test, incubating the antisera overnight with the homologous antigens for 12‐24 hours.^38^

Other antisera obtained from commercial sources were rabbit polyclonal anti‐GH (#AB940; Chemicon‐Merk‐Millipore, Schwalbach, Germany), guinea pig polyclonal anti‐PRL (#P9009‐16; United States Biological, Swampscott, MA, USA), mouse monoclonal anti‐human β‐LH (#L7500‐28B type Mab; United States Biological) and monoclonal mouse anti‐LEP (aa 131‐145; LS‐C25184/16340; LifeSpan BioSciences, Seattle, WA, USA). Polyclonal goat antibodies against different receptors (R) were purchased from Santa Cruz Biotechnology Inc.: anti‐LEPR (M‐18) (#sc‐1834), anti‐ACTHR (or anti‐MCR 2; #sc‐6879), anti‐FSHR (N‐20; #sc‐7798), anti‐LHR (K‐15; #sc‐26341), anti‐TSHR (N‐19; #sc‐7816); anti‐PRLR (E‐20; #sc‐21816) and anti‐GHR (L‐15; #sc‐10354).

### 2.3 Immunohistochemistry

Eight male mice, four WT and four KO, were deeply anaesthetised with a mixture of ketamine (100 mg kg^‐1^; Parke‐Davis, Alcobendas, Spain) and 2% xylazine (10 mg kg^‐1^; Dibapa, Barcelona, Spain) and transcardiacally perfused with 0.9% saline followed by fixation with Bouin's solution (0.9% picric acid, 9% formaldehyde, 5% acetic acid). The pituitary glands and abdominal adipose tissues were dissected out and postfixed by immersion in the same fixative for 36 hours. Subsequently, they were dehydrated in graded ethanol, cleared in xylene and embedded in paraffin. Horizontal sections, 3 μm in thickness, were cut on a microtome (Shandon Finesse 325; Thermo Electron Corporation, Waltham, MA, USA) and prepared for immunohistochemistry. Sections were collected consecutively onto slides to determine co‐localisations.

For the indirect immunohistochemical procedure, deparaffinised sections of abdominal adipose tissues were rehydrated in 0.05 mol L^‐1^ Tris buffered saline (TBS) (Trizma Base 0.05 mol L^‐1^, NaCl 0.9%) (pH 7.4), which was also used for all further incubations and washes. Sections were incubated overnight at room temperature with antibodies against OB/LEP (131‐145), OBR, ACTHR, FSHR, LHR, TSHR, PRLR or GHR (dilution 1:50). All antibodies were diluted in TBS buffer containing Triton‐100 at 0.2%. After rinsing, sections were incubated with biotinylated goat anti‐rabbit, goat anti‐mouse or rabbit anti‐goat antibodies (dilution 1:1000), followed by a streptavidin‐peroxidase conjugate (dilution 1:1000) (Jackson ImmunoResearch, West Grove, PA, USA), both for 60 minutes at room temperature. Peroxidase activity was detected using 0.004% 4‐chloro1‐naphthol as chromogen (Sigma Aldrich Co., Madrid, Spain) and 0.01% hydrogen peroxide. The specificity of the immunostaining was assessed by replacing the specific antiserum with normal serum, omitting one‐step of the reaction, or after preabsorption of the antiserum with the corresponding antigen.

To study the expression of DLK1, LEP and LEPR in relation to each pituitary cell producing hormones of the anterior lobe, an immunofluorescence procedure was carried out. To localise two or three markers on the same section, double‐ or triple‐immunofluorescence was used. Sections were first incubated overnight with a selected primary antibody diluted as described below, and then with the corresponding secondary antibody conjugated with a fluorophore for 1 hour at room temperature. The same procedure was followed with the second and the third primary antibodies. The dilutions used for antibodies were: ACTH^1^, ^2^, ^3^, ^4^, ^5^, ^6^, ^7^, ^8^, ^9^, ^10^, ^11^, ^12^, ^13^, ^14^, ^15^, ^16^, ^17^, ^18^, ^19^, ^20^, ^21^, ^22^, ^23^, ^24^, dilution 1:1000; hβFSH, dilution 1:1000; hβTSH, dilution 1:800; rPRL, dilution 1:800; guinea pig anti‐PRL, dilution 1:700; GH, dilution 1:2000; DLK1, dilution 1:1000; LEP (131‐145), dilution 1:50; and LEPR, dilution 1:50. For the case of goat antiserum against DLK1, sections were previously treated with citrate buffer (pH 6.0) at 90°C for 3 minutes. No differences in immunoreactivity were observed between the three anti‐DLK1 antisera.

Secondary antibodies used were Cy3‐conjugated anti‐rabbit; Cy2‐conjugated anti‐goat; Cy3‐conjugated anti‐mouse; DyLight 488‐conjugated anti‐guinea pig; Alexa 488‐conjugated anti‐rabbit; DyLight 405‐conjugated Streptavidin; and DyLight 649‐conjugated Streptavidin (dilution 1:1000 for all secondary antisera; Jackson ImmunoResearch).

Sections were examined and images acquired using a light microscope (DM4000B; Leica Microsystems, Wetzlar, Germany), digital camera (DFC300FX; Leica) and q‐win image analysis software (Leica, Barcelona, Spain). Only the image contrast was enhanced by linear adjustment. For immunofluorescence visualisation, a confocal microscope was used (DMI 4000B‐TCS SPE; Leica Microsystems) and images were processed with las‐af, version 2.6 (Leica).

### 2.4 Quantitative analysis

To study the different peptides and receptors in the pituitary of *Dlk1* ^−/−^ mice compared to controls, eight pituitaries were used: four from WT and four from KO mice. The pituitaries were cut in the horizontal plane to produce dorsoventral sections, 3 μm thick, situated 9 μm apart to ensure that the same cell was not counted twice. For each combination of markers, two sections per slide were measured for each pituitary. From each section, 10 fields were digitised at random. To establish the percentage of immunoreactive cells for each studied antigen, the number of immunopositive cells and the total number of cells were counted per unit area (30 000 μm²), visualising nuclei by 4′,6‐diamidino‐2‐phenylindole staining. To count cells, two researchers (RP‐A and MG‐G) worked in parallel counting the same set of sections, with strict accordance to discriminate single‐, double‐ or triple‐labelled cells from the background. Both researchers obtained similar counting results.

The percentages of GH, PRL, FSH, TSH and ACTH cells that colocalised with DLK1, LEP or LEPR were calculated, considering the number of cells immunoreactive to each hormone per unit area as 100%. A confocal microscope (DMI 4000B‐TCS SPE; Leica) was used to examine the sections, and cells were plotted and counted digitally using the Point and Multi‐point tools of imagej, version 1.43 (http://rsbweb.nih.gov/ij/download.html).

To quantitatively analyse LEP, LEPR and the receptors for the adenohypophyseal hormones ACTHR, TSHR, FSHR, LHR, PRLR and GHR, eight abdominal adipose tissue samples were used: four from WT and four from KO mice. Two sections per slide were selected, and 10 fields per section were captured at random.

Normal distribution was not found by Shapiro‐Wilk's test for small samples and statistical analysis was performed using a two‐tailed nonparametric Wilcoxon‐Mann‐Whitney test. *P*<.05 was considered statistically significant. All values are given as the mean±SEM.

### 2.5 RNA isolation and quantitative reverse transcriptase polymerase chain reaction (qRT‐PCR)

The levels of mRNA expression of the different hormone receptors in the abdominal adipose tissue or the pituitary gland were determined by qRT‐PCR. Tissues from five *Dlk1* WT and five KO male mice were rapidly removed after euthanasia, frozen in liquid nitrogen and maintained at −80°C until further use. Samples from five age‐matched male mice of each genotype were obtained for the study at the same time of the day to avoid fluctuations. They were crushed with liquid nitrogen in a sterile mortar and total RNA was isolated using the RNeasy kit (Qiagen, Madrid, Spain). Following DNase treatment with RNase‐Free DNase Set (Qiagen), 1 μg of RNA from each sample was reverse‐transcribed with the cDNA Revert Aid™HMinus First Strand cDNA synthesis kit (Fermentas, Madrid, Spain) in accordance with the manufacturer's instructions.

Analysis of gene expression was performed in a Step One Plus™ Real Time PCR System (Applied Biosystems, Foster City, CA, USA), in accordance with the Fast SYBR^®^ Green Protocol (Applied Biosystems). A final volume of 10 μL, including 4.2 μL of cDNA diluted 10 times in DNAase‐free water, 0.3 μmol L^‐1^ of each primer and 5 μL of Fast SYBR^®^ Green PCR Master Mix (Applied Biosystems), was analysed using the StepOne real‐time PCR detection system (Applied Biosystems). The PCR conditions used comprised an initial denaturation step at 95°C for 10 minutes, 40 cycles of 15 seconds at 95°C, followed by 1 minute at 60°C. The pairs of oligonucleotides specific for different mouse genes were designed using primerquest sm (Integrated DNA Technologies, Coralville, IA, USA) and are indicated in Table 1. All pairs of primers were validated and only one amplification product was obtained in all cases. The level of mRNA for the riboprotein P0 was used as an internal amplification control.^33^ To validate each pair of primers and assess amplification efficiency, standard curves were obtained with different cDNA dilutions from the pituitary. Amplification efficiency (*E*) was calculated according to the equation: *E* = 10^[−1/slope]^, where the slope was determined by a standard regression curve for each transcript. The mRNA levels were quantified relatively using the comparative Ct (cycle threshold) method 2^−∆∆Ct^,^39^ or the method of Pfaffl.^40^

**Table 1:** Mouse primers used for quantitative polymerase chain reaction amplifications

Gene	Forward primer	Reverse primer
*PO*	5′‐AAGCGCGTCCTGGCATTGTCT‐3′	5′‐CCGCAGGGGCAGCAGTGG T‐3′
*Acthr*	5′‐GCCATTTCTGACATGTTGGGCAGT‐3′	5′‐AGGCTGAAGATAGAGCCCAGCAAA‐3′
*Fshr*	5′‐ACAACTGTGCATTCAACGGAACCC‐3′	5′‐ATGGTTGGGCAGGGAATAGACCTT‐3′
*Ghr*	5′‐AGCCTCGATTCACCAAGTGTCGTT‐3′	5′‐CAGCTTGTCGTTGGCTTTCCCTTT‐3′
*Lhr*	5′‐CAATGCAGTGGCCTTTGTCGTCAT‐3′	5′‐TTGTGTCCTTGTTAGGAGCCGTCA‐3′
*Lep*	5′‐AGCAGTGCCTATCCAGAAAGTCCA‐3′	5′‐AATGAAGTCCAAGCCAGTGACCCT‐3′
*Lepr*	5′‐AAACAATGCCTCGGCTTTGAAGGG‐3′	5′‐TGCTCATTCCCAAAGCAACAGTGG‐3′
*Prlr*	5′‐TGGATCATTGTGGCCGTTCTCTCT‐3′	5′‐TCAGCAGTTCTTCAGACTTGCCCT‐3′
*Tshr*	5′‐ACTCCTGTGCCAATCCGTTTCTCT‐3′	5′‐GCCAAACTTGCTGAGCAGGATGAA‐3′

*PO*, ribosomal phosphoprotein gene; *Acthr*, adrenocorticotrophic hormone receptor gene; *Fshr*, follicle‐stimulating hormone receptor gene r; *Ghr*, growth hormone receptor gene; *Lep*, leptin gene; *Lepr*, leptin receptor gene; *Lhr*, Luteinising hormone receptor gene; *Prlr*, prolactin hormone receptor gene; *Tshr*, thyroid stimulating hormone receptor gene.

Statistical data for each target gene were determined in five different animals, each carried out in triplicate. The number of animals required for the studies was calculated using granmo (http://www.imim.cat/ofertadeserveis/software-public/granmo/). Accordingly, accepting an α risk of 0.1 and a β risk of 0.2 in a two‐sided test, five subjects were required in each group to recognise a difference ≥1.9 units as being statistically significant. The common SD was assumed 1.2. A drop‐out rate of 0% was anticipated. Statistical analysis was performed using a two‐tailed nonparametric Wilcoxon‐Mann‐Whitney test; normal distribution was not found by Shapiro‐Wilk's test for small samples. *P*<.05 was considered statistically significant.

## 3 RESULTS

In the present study, we tested immunohistochemically for the presence of receptors for hormones released by cells of the two tissues in WT 129/Svj mice (WT; *Dlk1* ^+/+^) compared to KO mice (*Dlk1* ^−/−^) to link the presence of DLK1 protein with the other molecules under consideration. The mRNA expression of these molecules was also determined by qRT‐PCR.

### 3.1 Expression of adenohypophyseal hormone receptors in the adult abdominal adipose tissue of WT male mice

**Figure 1:**
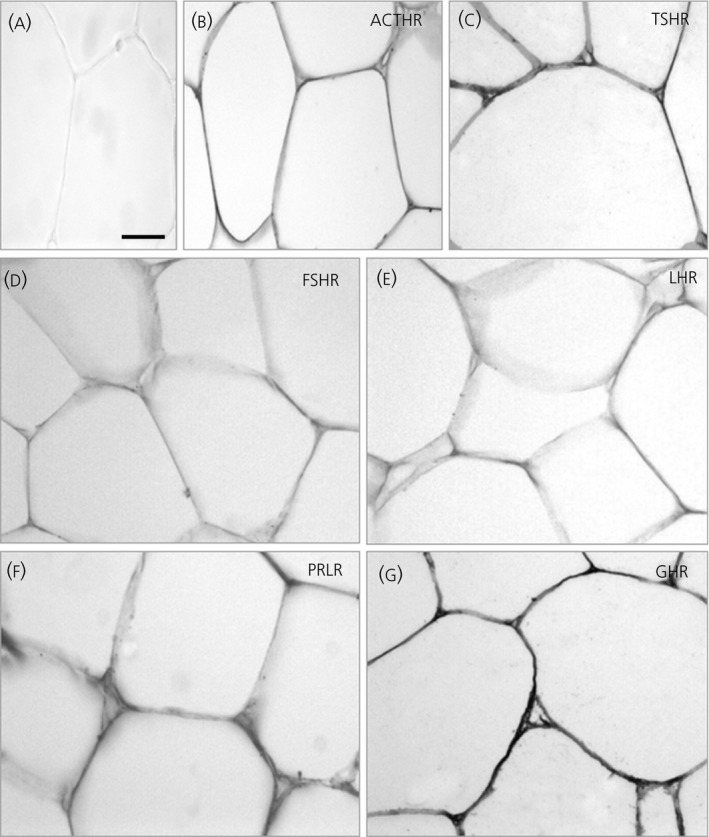
Immunohistochemical detection of receptors for adenohypophyseal hormones in adipocytes of wild‐type male mice. (A) Negative control showing absence of immunoreaction. (B) Adrenocorticotrophic hormone receptor (ACTHR). (C) Thyroid‐stimulating hormone receptor (TSHR). (D) Follicle‐stimulating hormone receptor (FSHR). (E) Luteinising hormone receptor (LHR). (F) Prolactin hormone receptor (PRLR). (G) Growth hormone receptor (GHR). For detection of peroxidase, 4‐chloro‐1‐naphthol was used as chromogen. Scale bar=10 μm

**Figure 2:**
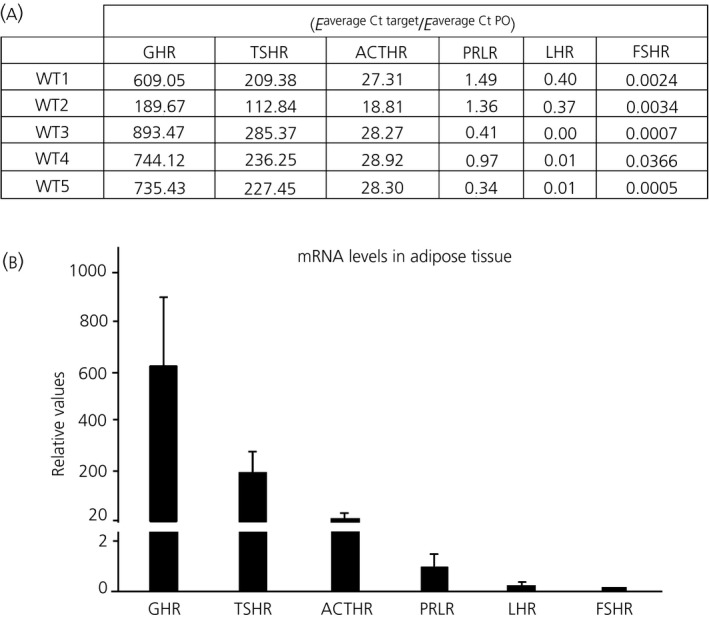
(A) Relative values in arbitrary units of mRNA expression of adenohypophyseal hormone receptors in adult abdominal/white adipose tissue of five wild‐type (WT) mice (WT1‐WT‐5), quantified using the method of Pfaffl,^40^ with the riboprotein P0 analysed as the internal control.^40^ Each value is the average of a triplicate experiment. (B) Graphic representation of the values indicated in (A), arranged according to the expression levels. Each column represents the mean±SEM of the mRNA expression in the five WT animals. ACTHR, ACTH receptor; Ct, threshold cycle values; E, efficiency of oligonucleotides; FSHR, follicle‐stimulating hormone receptor; GHR, growth hormone receptor; LHR, luteinising hormone receptor; PO, ribosomal phosphoprotein gene; PRLR, prolactin receptor; TSHR, thyroid‐stimuating hormone receptor

Adipocytes were immunoreactive for all specific antibodies against adenohypophyseal hormone receptors ACTHR, TSHR, FSHR, LHR, PRLR and GHR (Figure 1B‐G). The mRNA expression of these receptors in the abdominal adipose tissue of adult mice was subjected to qRT‐PCR, using specific oligonucleotides, and analysed with the method of Pfaffl.^40^ The data indicated that the receptors with the highest RNA expression were GHR and TSHR, followed by ACTHR, whereas PRLR, LHR and FSHR had a lower level (Figure 2). In all cases, high variability among samples was observed.

### 3.2 Expression of the LEPR in the anterior pituitary lobe and abdominal adipose tissue of WT male mice

**Figure 3:**
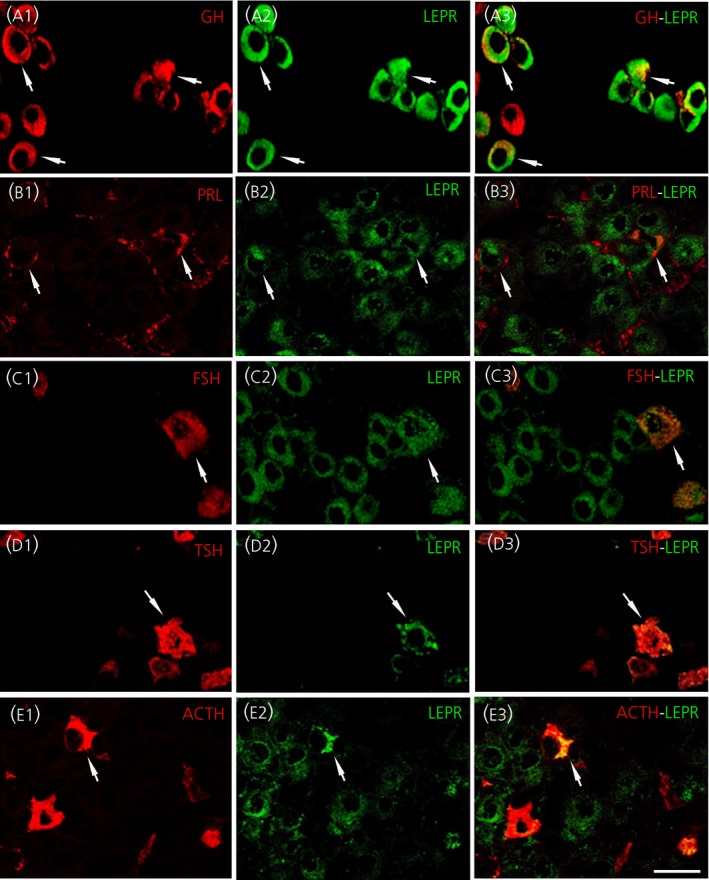
Immunohistochemical detection of adenohypophyseal hormone‐producing cells that express the leptin receptor (LEPR) in adult wild‐type mice. Horizontal pituitary sections were stained with the double immunofluorescence method. The first column shows each of the hormone‐producing cells (red) in the pituitary anterior lobe. (A1) Growth hormone (GH). (B1) Prolactin (PRL). (C1) Follicle‐stimulating hormone (FSH). (D1) Thyroid‐stimuating hormone (TSH). (E1) Adrenocorticotrophic hormone (ACTH). The second column (A2‐E2) shows leptin‐immunoreactive cells (LEPR‐IR; in green). The third column (A3‐E3) shows double‐labelled cells. The arrows point to conspicuous double‐labelled cells. Scale bar=10 μm

LEPR‐immunoreactive (‐IR) cells were widely distributed throughout the anterior pituitary lobe (see Supporting information, Figure S1A), constituting 17.09±0.9% of cells. The double immunofluorescence staining method showed that cell types producing pituitary hormones were also LEPR‐IR (41.5±3.8% of GH‐IR cells, 13.5±1.7% of PRL‐IR cells, 3.5±1.3% of FSH/LH‐IR cells, 3.3±0.6% of TSH‐IR cells and 3.0±0.5% of ACTH‐IR cells) (Figure 3A1‐3,B1‐3,C5‐3,D1‐3,E1‐3, respectively).

**Figure 4:**
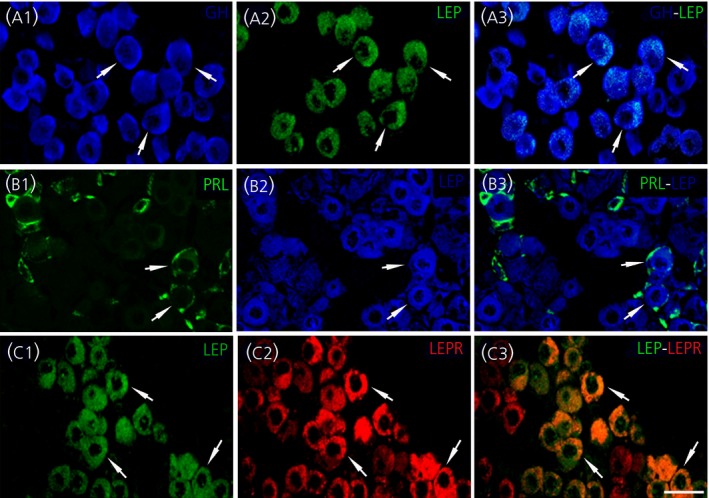
Expression of the adipocyte leptin (LEP) in somatotroph and lactotroph cells, and expression of leptin receptor (LEPR) in LEP‐immunoreactive (IR) cells, in horizontal sections of the pituitary anterior lobe of adult male mice, as detected with a double‐stained immunofluorescent method. (A1‐A3) Colocalisation of LEP‐IR (green) and growth hormone (GH)‐IR (blue) in somatotroph cells. (B1‐B3) Colocalisation of LEP‐IR (blue) and prolactin (PRL)‐IR (green) in lactotroph cells. (C1‐C3) LEP‐IR cells (green) that are LEPR‐IR (red) are shown in C3. The arrows point to conspicuous double‐labelled cells. Scale bar=10 μm

In addition to LEPR‐IR cells, immunoreactive to leptin (LEP‐IR) were seen in the anterior lobe (see Supporting information, Figure S1C), comprising 6.8±1.1% of total cells. Double immunostaining showed that only somatotroph and lactotroph cells were LEP‐IR, involving 15.8±3.0% and 9.6±1.3%, respectively (GH‐ LEP‐IR and PRL‐ LEP‐IR) (Figure 4A1‐3,B1‐3). Moreover, all LEP‐IR cells were also LEPR‐positive (LEP‐ LEPR‐IR) (Figure 4C1‐3). LEP expression by qRT‐PCR was almost undetectable in the pituitary gland in all samples, whereas the LEPR mRNA levels were somewhat higher but also low (not shown).

**Figure 5:**
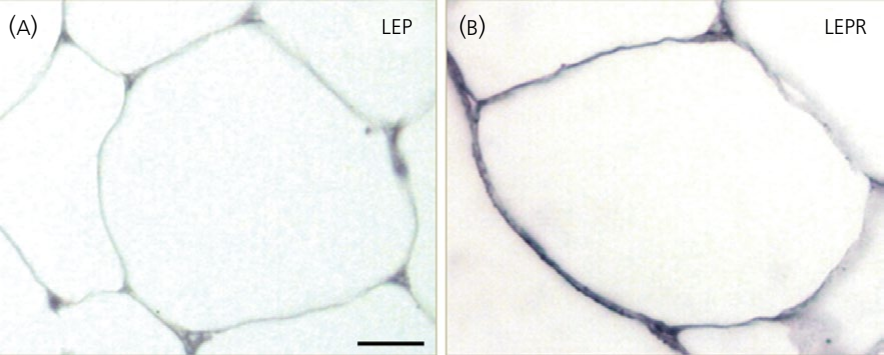
Expression of leptin and its receptor in adipose cells. Immunohistochemical detection of the adipocyte leptin (LEP) hormone (A) and its receptor (LEPR) (B) in adipocytes of adult male mice. Note that LEP‐immunoreactivity is not strong because leptin is quickly released.^60^ Scale bars: (A, B)=10 μm.

In LEP‐producing adipocytes, immunoreactivity against their LEPR receptor was also observed (Figure 5). The LEPR mRNA expression was very low and similar between all the adipose tissue samples, whereas LEP expression as determined by qRT‐PCR was significantly higher but with an elevated variability between samples (Table 2).

**Table 2:** Relative values of mRNA expression of leptin (LEP) and its receptor (LEPR) in abdominal adipose tissue of five wild‐type mice, according to the method of Pfaffl^40^

	(*E* ^average Ct target^/*E* ^average Ct PO^)	
	LEP	LEPR
WT1	20 125	0.089
WT2	2271	0.059
WT3	55 613	0.055
WT4	65 664	0.131
WT5	168 092	0.085

Ct, threshold cycle values; *E*, efficiency of oligonucleotides; PO, ribosomal phosphoprotein gene; WT, wild‐type.

### 3.3 Immunohistochemical analysis of the DLK1 protein with respect to LEP, LEPR, and GH and PRL adenohypophyseal hormones in WT mice

In a previous study, we demonstrated that DLK1 protein is present in pituitary cells, mainly GH and PRL cells.^33^ Recently, we found that 70±5.9% GH‐IR cells, 49.3±3.0% PRL‐IR cells, 11.0±4.6% FSH/LH‐IR cells and 6.7±1.7% ACTH‐IR cells expressed DLK1.^41^

**Figure 6:**
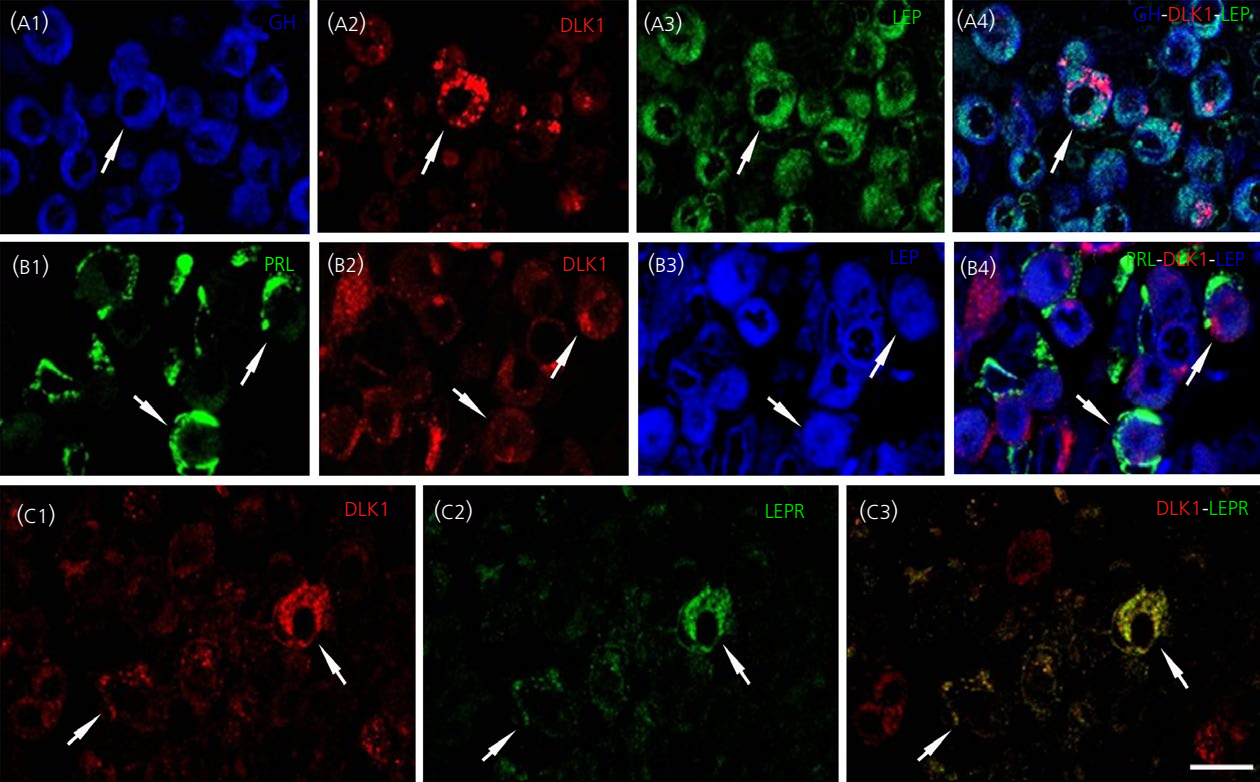
Detection of delta‐like protein 1 (DLK1) in cells of the anterior pituitary lobe of adult male mice using a double‐ or triple‐stained immunofluorescent method. (A1‐A3) Growth hormone (GH)‐immunoreactive (‐IR) cells (blue) that are DLK1‐IR (red) and lepton (LEP)‐IR (green). (A4) Merged image of the three dyes. (B1‐B3) Prolactin (PRL)‐IR cells (green) that are DLK1‐IR (red) and LEP‐IR (blue). (B4) Merged image of the three dyes. (C1‐C3) Expression of the leptin receptor LEPR (green) in DLK1‐IR cells (red) in selected horizontal sections of pituitary (C3) Merged image of the two markers. Arrows point to cells that express two or three markers. Scale bar=10 μm

The immunochemical tests determined that 16.1±2.6% of DLK1‐IR cells were also LEP‐IR (DLK1‐LEP‐IR cells) (Figure 6A2‐4,B2‐4). Moreover, triple immunofluorescence revealed that 6.8±1.2% of GH‐IR and 6.0±0.8% of PRL‐IR cells were also DLK1‐ LEP‐IR, (Figure 6A1‐4,6,B1‐4). Testing those DLK1‐IR cells presenting LEPR showed that 46.6±1.2% of DLK1‐IR cells were also LEPR‐IR (DLK1‐ LEPR‐IR cells) (Figure 6C1‐3).

### 3.4 Expression of adenohypophyseal hormone receptors in the abdominal adipose tissue of male mice deficient in the *Dlk1* gene

In adipocytes from *Dlk1* ^−/−^ mice, the presence of immunoreactive cells was detectable for all antibodies used against adenohypophyseal hormone receptors (ACTHR, TSHR, FSHR, LHR, PRLR and GHR) (not shown).

**Figure 7:**
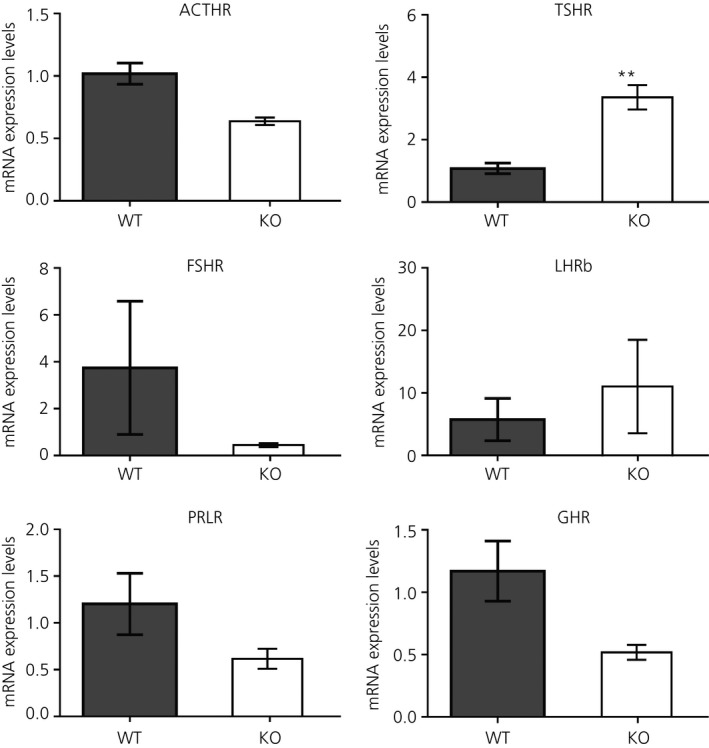
mRNA levels of each adenohypophyseal hormone receptor in the abdominal adipose tissue of Dlk1^−/−^ male mice [knockout (KO); n = 5] compared to controls [wild‐type (WT); n = 5]. Note that a significant increase is only detected in thyroid‐stimulating hormone receptor (TSHR) mRNA levels in KO mice related to WT. Each column represents the mean±SEM of mRNA levels in WT and KO animals. **Statistically significant difference vs control animals (*P*<.01). ACTHR, adrenocorticotrophic hormone receptor; FSHR, follicle‐stimuating hormone receptor; GHR, growth hormone receptor; LHRb, luteinising hormone receptor; PRLR, prolactin receptor; TSHR, thyroid‐stimulating hormone receptor

The expression of ACTHR, TSHR, FSHR, LHR, PRLR and GHR was also analysed by qRT‐PCR in KO compared to WT mice (Figure 7). The mRNA levels of the hypophyseal hormone receptors were similar to those of WT mice, except for a significant increase (*P*<.01) in TSHR mRNA expression in *Dlk1* KO mice (Figure 7).

### 3.5 Presence of the specific LEPR in adenohypophyseal cells and abdominal adipose tissue from *Dlk1* KO mice

In *Dlk1* KO mice, LEPR‐IR cells were also observed in the anterior lobe of the adenohypophysis (see Supporting information, Figure S1B). In this case, the percentage of LEPR‐IR cells was 27.17±1.5%, which is significantly higher than in WT controls (17.09±0.9%; *P*≤.0001) (Table 3). As in WT mice, all adenohypophyseal cell types in mutant mice were LEPR immunoreactive, although in different proportions (Table 3). The somatotroph cells showed the highest percentage (53.1±4.0% LEPR‐IR vs 41.5±3.8% in controls with *P *≤ .05). No significant differences in the other hormone‐producing cell types were found between controls and *Dlk1* KO mice. Therefore, only the GH‐LEPR‐IR cell type presented significant differences with respect to WT mice (Table 3).

**Table 3:** Presence of leptin (LEP) and its leptin receptor (LEPR) in the hormone‐producing cells of the pituitary anterior lobe in knockout (KO) and wild‐type (WT) mice

Cell type	% Of cells	
	KO	WT
LEPR‐IR	27.17±1.5***	17.09±0.9
GH‐LEPR‐IR	53.1±4.0*	41.5±3.8
PRL‐LEPR‐IR	16.1±1.6	13.5±1.7
FSH‐LEPR‐IR	5.0±0.9	3.5±1.3
TSH‐LEPR‐IR	4.0±0.8	3.3±0.6
ACTH‐LEPR‐IR	3.6±0.7	3.01±0.5
LEP‐IR	3.0±0.6*	6.8±1.1
GH‐LEP‐IR	10.2±1.7	15.8±3.0
PRL‐LEP‐IR	4.8±1.1**	9.6±1.3
LEP‐LEPR‐IR	100	100

Data are presented as the mean±SEM.

KO, knockout; WT, wild‐type.ACTH, adrenocorticotrophic hormone; FSH, follicle‐stimulating hormone; GH, growth hormone; ‐IR, immunoreactive; PRL, prolactin; TSH, thyroid‐stimulating hormone.

*Significant difference vs WT control mice with *P*<.05. **Significant difference vs WT control mice with *P*<.01. ***Significant difference vs WT control mice with *P*<.001.

Few LEP‐IR cells were observed in the anterior pituitary lobe of KO mice (see Supporting information, Figure S1D), comprising 3.0±0.6% with respect to the total, as opposed to 6.8±1.1% of LEP‐IR cells in WT mice (Table 3). Similar to controls, only somatotroph and lactotroph mouse cells were immunoreactive to LEP (10.2±1.7% of the somatotroph‐LEP‐positive cells and 4.8±1.1% of the lactotroph LEP‐IR cells). This was in comparison with 15.8±3.0% and 9.6±1.3%, respectively, in WT controls, with the differences being statistically significant in the lactotroph cells (*P *≤ .01) with respect to WT controls, whereas nonsignificant differences were found in the somatotroph cells (Table 3). By contrast, all LEP‐IR cells were also LEPR‐IR, as in controls (Table 3).

Finally, LEP mRNA was highly expressed in all abdominal white adipose samples, whereas LEPR mRNA levels were quite low, although no significant differences between KO and WT mice were found in any case (see Supporting information, Figure S1, bottom).

## 4 DISCUSSION

**Figure 8:**
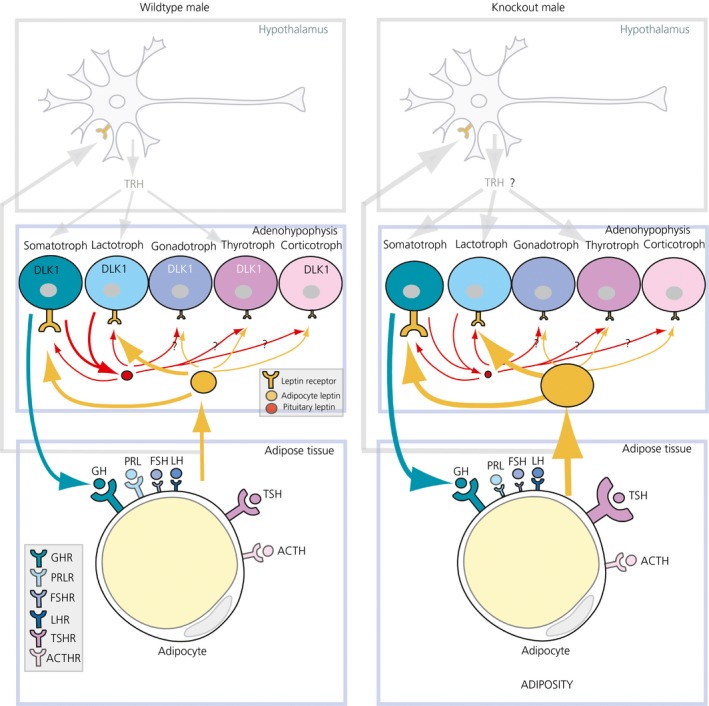
Schematic representation of proposed relationships between adenohypophyseal hormone‐producing cells and adipocytes in the adult male mouse, anddelta‐like protein 1 (DLK1) involvement in them. Left: representative cells of the hypothalamic‐pituitary‐adipose axis and its hormonal interactions in the male wild‐type mouse, strain 129/SvJ. Top square represents a thyroid‐releasing hormone (TRH)‐expressing neurone in the hypothalamus with leptin receptor (LEPR) (orange); its interactions with pituitary and adipose cells are represented in grey. Middle square symbolises the adenohypophysis with the five main hormone‐producing cells (somatotroph, lactotroph, gonadotroph, thyrotroph, corticotroph), distinguished by a specific colour. Although all of these cell types express DLK1, this protein is mainly present in growth hormone (GH) and prolactin (PRL) cells (DLK1 in a larger font), which are the only ones producing leptin in the adenohypophysis (thick red arrows and red circle). DLK1 is thus implicated in pituitary LEP expression. Leptin receptor LEPR (orange) is expressed by each of the adenohypophyseal cells, mainly by GH and PRL cells; the size of the LEPR symbol is related to its relative abundance in each of the adenohypophyseal cells. Pituitary leptin (red circle) may act on adenohypophyseal cells through their LEPR (narrow red arrows with question marks), more probably on GH and PRL cells and potentially on follice‐stimulating hormone (FSH)/luteinising hormone (LH), thyroid‐stimuating hormone (TSH) and adremocorticotrophic hormone (ACTH) cells (question marks in narrow red arrows). Hormones GH, PRL, FSH, LH and TSH act on adipose cells (coloured arrows associated with the hormone‐producing cell). The bottom square represents a simplification of abdominal adipose tissue, with a single adipose cell. Adipocytes express receptors for GH, PRL, FSH, LH, TSH and ACTH hormones released by adenohypophyseal cells; the size of each hormone receptor symbol expresses the relative presence of each of them in adipocytes. These hormones, particularly GH, affect adipocyte leptin secretion. Adipose cells produce abundant leptin (orange circle) that acts on adenohypophyseal cells through LEPR, regulating GH and PRL cells (thick orange arrows) and the other adenohypophyseal cells (thin orange arrows). Right: Main changes found in the pituitary‐adipose axis in male *Dlk1* ^−/−^ KO mice. Directly or indirectly, *Dlk1* deficit leads to a lower number of GH cells and increased leptin levels in serum^33^ a decrease in pituitary leptin in GH and PRL cells, and LEPR increase in GH cells. In adipose cells, TSH receptor (TSHR) and LH mRNA levels are raised, whereas PRL mRNA levels are lowered. A significant increase in mRNA of TSHR in adipocytes is related to adiposity through increased adipose leptin action on hypothalamic TRH‐releasing neurones by increasing TRH release (question mark ‘?’). ACTHR, ACTH receptor; FSHR, FSH receptor; GHR, GH receptor; LHR, LH receptor; PRLR, PRL receptor

In the present study, we have analysed the impact of the lack of DLK1 with respect to the expression of adenohypophyseal hormone receptors in the adipose cells from abdominal adipose tissue, as well as of adipocyte LEPR in adenohypophyseal cells. In the 129/Svj male mouse, DLK1 is a recognised modulator of both adipogenesis and neuroendocrine axes. With this aim, a KO mouse deficient in DLK1 protein was used. Dlk1 KO mice have more visceral adipose tissue but a reduced body size, fewer GH cells and increased serum LEP levels.^33^, ^36^ In the present study, we show how DLK1 has a certain role in the interactions between the adipose tissue and the adenohypophyseal cells; this is particularly the case in GH‐adipose cells. Moreover, DLK1 can directly or indirectly mediate in pituitary LEP production in GH and PRL cells. Besides this, Dlk1 KO mice exhibited an altered expression of the TSH receptors in adipocytes (Figure 8).

In the 129/Svj mouse, receptors for all adenohypophyseal hormones were detected in the adipose cells in vivo. In a previous in vitro study, expression of receptors for the pituitary hormones (except FSHR and LHR) was detected in cultured adipose cells.^5^ Later, FSHR was detected at protein and mRNA level in adipocytes of abdominal adipose tissue in female chickens.^6^ As in other vertebrate groups,^14^, ^16^, ^18^ we have confirmed that all adenohypophyseal cells in the 129/Svj male mouse contain LEPR, including gonadotrophs. In previous studies, LEPR was found in folliculostellate cell lines and in some pituitary cell types of female mice (nonspecified strains), as well as in TSH cells of female rat pituitary tissue,^15^ or in all adenohypophyseal cell types using dispersed and cultured cells of female mouse pituitaries.^9^ These findings confirmed the relationship between pituitary and adipose cells and, consequently, numerous physiological studies were developed to demonstrate the interaction of LEP with the different pituitary axes. This was necessary because pituitary hormone secretion is highly dependent on the metabolic status of the organism.^8^, ^41^, ^42^, ^43^, ^44^ In particular, many studies have focussed on the regulatory role of LEP on GH, noting reductions in GH expression and circulating GH in leptin‐deficient obese^8^ and LEPR‐KO mice.^9^ The location of LEPR and LEP in GH cells is well documented,^15^, ^18^, ^21^, ^45^ suggesting a potential autocrine action of LEP on these cells. Our data for the 129/Svj male mouse indicate that GH cells express the highest percentage of LEPR (41.5%). Moreover, GH cells together with PRL were the only adenohypophyseal cells co‐expressing LEP. This specific cell location of LEP in the male pituitary appears to be specific to this mouse strain. According to previous studies, detection of LEP in pituitary anterior lobe cells indicated great dissimilarities between the species studied. In rats, LEP was first located mainly in TSH cells.^15^ However, GH cells were recognised as the main cells producing LEP in a study using dispersed and cultured cells from rat pituitaries, with different percentages in females according to the stage of the oestrous cycle.^20^ Later, using the same method, Akhter et al^23^ also demonstrated the presence of LEP mRNA in gonadotroph cells in male and female rats. In a recent review, it was suggested that specifically pituitary LEP (mainly located in GH cells) is necessary to maintain GH and LH functions. Previously, the same group reported LEP expression in all adenohypophyseal cells in Sprague‐Dawley rats, largely in GH cells, under both normal and fast conditions.^46^ It was found that 84% of GH cells co‐expressed LEP, whereas only 0.6% of PRL cells contained LEP. In humans, LEP was located by electron microscopy in secretory granules of all adenohypophyseal cells (principally ACTH cells), except PRL cells.^21^ This nondetection of LEP in PRL cells may be related to electron microscopy characteristics. Indeed, other groups found LEP in all adenohypophyseal cell types in humans, most frequently in ACTH cells.^22^, ^47^ However, in the 129/Svj mouse, GH and PRL cells were the only types producing LEP. Because LEP is an essential hormone in metabolic regulation, with great functional plasticity in different metabolic situations,^23^, ^48^ this regulation may change depending on species, strain and age, as well as reproductive and/or nutritional stage. Therefore, different observations regarding the cellular location of pituitary LEP are clearly possible. Another factor explaining the differences in published results is the variability in the methods used.

It is known that in vivo techniques may involve difficulties because of the tissue processing, although in vitro cultured cells, representing the only studies performed in mice to date, also present problems that need to be considered. In dispersed cultured cells, molecular expression can be different as a result of the environment and natural context of cell interactions being altered. Although observed differences in cell types may not be a consequence of this, they can affect the dissimilarities in the reported percentages.

The potential role of LEP in GH cells has been studied using KO mice for the adipocyte or pituitary LEP,^11^, ^12^ a deletion of exon 17 of *LepR* in somatotroph cells^9^ and LEPR deletion in GH cells.^49^ Several interesting findings arose from these studies. First, adipose cells are the main circulating LEP source with metabolic activity and, second, pituitary LEP mainly affects expression in GH cells and their cell number. Accordingly, a reduction in GH activity and an increase in adiposity were observed. Interestingly, a dramatic reduction was found in serum PRL in somatotroph‐*Lep* lacking mice, suggesting an important action of pituitary LEP in the maintenance of GH and PRL cell types.^12^ However, according to the data, the serum PRL reduction detected in these mutants was restricted to 6‐month‐old female mice, and was not found in 21‐day‐old female or male mice. Noteworthy, our previous results with the male KO mouse deficient in DLK1 protein showed a lower number of GH cells and augmented adiposity, although GH secretion apparently persisted because no changes in serum GH levels were detected.^33^ In the present study, using the same animal model, pituitary LEP decreased in the GH and PRL cells, together with a significant increase in LEPR, specifically in GH cells. These findings suggest that DLK1 protein could be necessary for pituitary LEP expression in GH and PRL cells. However, the LEPR increase in GH cells of Dlk1‐deficient mice (present results) coincides with the LEP increase in serum previously shown in equal KO mice.^33^ This is adipocyte leptin according to Odle et al.^12^ These results would confirm that both LEP sources are necessary for correct GH activity. All of these observations point to a possible feedback between adipose and GH cells.

Additionally, GH hormone can also block preadipocyte differentiation by induction of *Dlk1* expression,^50^ which is considered to be a modulator of adipogenesis (see Introduction). Consequently, the increased adult adiposity observed in our mutant model may be influenced directly by the absence of DLK1 because this protein also acts on other factors proposed as promotors of adiposity, such as LEP, GH or TSHR (see below).

No variations in GHR expression with respect to adenohypophyseal hormone receptors were observed in adipose cells, although, in our model, there was a significant LEPR increase in GH cells. Nevertheless, in the adipose cells of this *Dlk1* ^−/−^ mutant mouse, a significant rise was only found in the TSHR, suggesting that DLK1 participates directly or indirectly in the action of TSH on adipocytes, which needs to be tested. Indeed, altered expression of TSHR could lead to increased adipogenesis.^51^ An increase was found in TSHR expression at mRNA or protein level in adipocyte differentiation, in visceral adipose tissues from obese mice and in s.c. adipose tissue from humans with higher body‐mass index. Despite the role of TSH on adipose cells being unclear so far, recent studies have demonstrated that this hormone induces an inhibition of the adipose triglyceride lipase enzyme through the TSHR receptor in cultured adipocytes,^52^ as well as an increase in triglyceride storage.^53^ Both findings are probably correlated with augmented adiposity. The higher LEP levels in serum, also observed in our KO mouse model,^33^ might act on the hypothalamus to increase thyroid‐releasing hormone (TRH) expression through LEPR.^54^ TRH is not only a releasing factor for TSH, but also for GH and PRL hormones.^55^ In our mouse model lacking *Dlk1*, serum GH levels were slightly higher than in the WT male mouse, whereas PRL cells contained less immunoreactivity.^33^ This lower immunoreactivity does not appear to be related to higher PRL release because PRL levels in serum were practically undetectable^33^ and some reduction in receptors for this hormone in adipose cells was observed in mutant mice (present study). PRL has a role not only in preadipocyte differentiation, but also in the metabolism of differentiated adipocytes by increasing LEP levels.^56^ However, in our model, PRL does not appear to be the hormone responsible for augmented LEP levels, at least directly, suggesting an indirect action.

The hormone LEP also has an important action on the reproductive system and “may act as the critical link between adipose tissue and the reproductive system, indicating whether adequate energy reserves are present for normal reproductive function”.^7^ In the pituitary, mice lacking DLK1 protein express significantly less FSH mRNA without showing changes in LH mRNA.^33^ These results suggest a difference in the role of DLK1 in the synthesis of these hormones. Furthermore, abdominal adipose tissue shows a nonsignificant increase in LHR expression and a lower FSHR expression level according to the results of the present study. Indeed, published data on the rat indicate that LEP indirectly regulates hypothalamic GnRH production under normal conditions and directly stimulates the production of gonadal hormones by LEPR, expressed in gonadotroph cells.^57^ In sheep and rat pituitaries, gonadotrophs were found to be the main type of adenohypophyseal cells expressing LEPR.^16^, ^18^ This suggests that LEP produced by gonadotroph cells acts in a paracrine manner on GH cells in these two species. Moreover, Akhter et al^58^ found that a percentage of dispersed rat pituitary cells in cell cultures were also gonadotrophs, although most adenohypophyseal cells were also GH immunoreactive. The GnRH hormone was considered to be the factor responsible for pituitary LEP, with a high plasticity in LEP expression being observed through the female cycle. In our model, 3.5% of WT gonadotroph cells expressed LEPR but not LEP. However, LEP is present in PRL cells, which have a documented relationship with gonadotroph cells.^59^ Moreover, DLK1 could also exert some juxtacrine signalling effects on gonadotroph cells.^33^ However, in *Dlk1* ^−/−^ mice, there was a nonsignificant LEPR increase in gonadotroph cells, together with a significant reduction in LEP in PRL cells. This suggests that adipose LEP acts on gonadotroph cells.

In summary, although physiological studies are needed, the results of the present study suggest that DLK1 is a factor to be considered not only as a modulator of adipocyte differentiation, but also of the differentiated adipocyte function. It may thus be an important factor in the relationships between adipose tissue and the pituitary hormone producing cells. Therefore, the observed adiposity in *Dlk1‐*deficient mice may be a consequence of DLK1 being involved in the different feedbacks between adipose LEP and the production of pituitary hormones. Hormones GH, PRL, LH and TSH all act on the adipose cells, stimulating or inhibiting LEP synthesis and release, inhibiting lipase, and increasing triglyceride storage or the number of adipose cells. In addition, DLK1 appears to directly or indirectly modulate pituitary LEP synthesis, particularly in PRL cells. Figure 8 presents tentatively proposed interactions between adenohypophyseal cells and adipocytes based on previous and present results, which require further in vitro physiological tests.
