# Supplementary figures and images for: Delta‐like protein 1 in the pituitary‐adipose axis in the adult male mouse
Source: J Neuroendocrinol. 2017 Aug 28;29(8):e12507. doi: 10.1111/jne.12507 (PMC6084355; doi:10.1111/jne.12507)

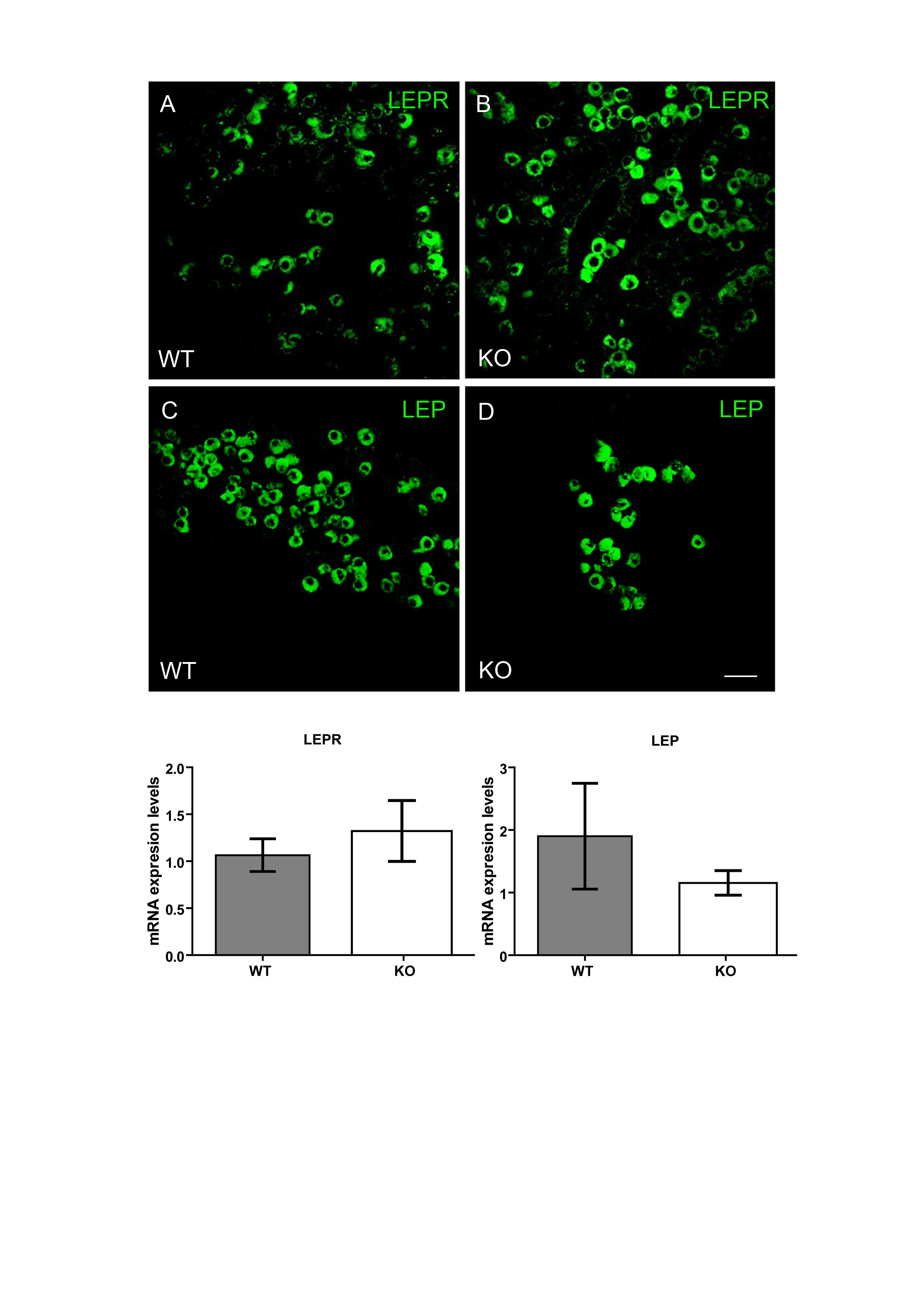

Supplement: Supplementary file 1 [file JNE-29-na-s001.tif]
