# Supplementary material for: Delta‐like protein 1 in the pituitary‐adipose axis in the adult male mouse
Source: J Neuroendocrinol. 2017 Aug 28;29(8):e12507. doi: 10.1111/jne.12507 (PMC6084355; doi:10.1111/jne.12507)
Supplement: Supplementary file 2 [file JNE-29-na-s002.docx]

**Supplemental Table 1**. Primary antibodies used in this work

| **Peptide/Protein Target** | **Antigen Sequence** | **Name of Antibody** | **Source** | **Species Raised** |
| --- | --- | --- | --- | --- |
| ACTH | Synthetic ACTH (amino acids 1-24)  (CIBA, Basel, Switzerland) | Anti-ACTH (1-24) | G. Tramu (University of Bordeaux 1, Bordeaux, France) | Rabbit polyclonal |
| ACTHR | Epitope mapping at the C-terminus of MC2-R of human origin | MC2-R (melanocortin 2 receptor) Antibody (C-16) | sc-6879; Santa Cruz Biotechnology Inc., Germany | Goat polyclonal |
| DLK1 | 217 amino acids of mouse DLK1 corresponding to the six EGF-like repeats of its extracellular region | Anti-DLK1 (#1125) | Laboratory of J. Laborda (University of Castilla-La Mancha; Albacete, Spain) | Rabbit polyclonal |
| DLK1 | C-terminus of DLK of human origin | Anti-DLK (C-19) | sc-8624; Santa Cruz Biotechnology Inc., Germany | Goat polyclonal |
| DLK1 | Amino acids 266-383 mapping at the C-terminus of DLK of human origin | Anti-DLK (H-118) | sc-25437; Santa Cruz Biotechnology INC., Germany | Rabbit polyclonal |
| FSH | Human βFSH (Chemicon International, Los Angeles, CA, USA) | Anti-hβFSH | G. Tramu (University of Bordeaux 1, Bordeaux, France) | Rabbit polyclonal |
| FSHR | Epitope mapping near the N-terminus of FSHR of human origin | FSHR Antibody (N-20) | sc-7798; Santa Cruz Biotechnology INC., Germany | Goat polyclonal |
| GH | Purified growth hormone | Anti-Growth Hormone | AB940; Chemicon-Merk-Millipore; Schwalbach, Germany | Rabbit polyclonal |
| GHR | Epitope mapping near the N-terminus of GHR of mouse origin | GHR Antibody (L-15) | sc-10354; Santa Cruz Biotechnology Inc., Germany | Goat polyclonal |
| LEP | Amino acid 131-145 of human leptin | Mouse anti-Human Leptin (LEP) | LS-C25184/16340; LifeSpan Biosciences Inc.; Seattle, WA, USA | Mouse monoclonal |
| LEPR | Epitope mapping at the C-terminus of the short form of Ob-R (LEPR) of mouse origin | Ob-R Antibody (M-18) for detection of short and long forms of OBR (LEPR) | sc-1834; Santa Cruz Biotechnology Inc., Germany | Goat polyclonal |
| LH |  | Anti-Luteinizing Hormone beta (LH) | L7500-28B; United States Biological (Swampscott, MA, USA) | Mouse monoclonal |
| LHR | Peptide mapping within an internal region of LHR of human origin. | Anti LHR (K-15) | sc-26341; Santa Cruz Biotechnology Inc., Germany | Goat polyclonal |
| PRL | Rat PRL (Chemicon International, Los Angeles, CA, USA) | Anti-rPRL | G. Tramu (University of Bordeaux 1, Bordeaux, France) | Rabbit polyclonal |
| PRL |  | Anti-Prolactin (PRL) Pab Gp xHu | P9009-16; United States Biological (Swampscott, MA, USA) | Guinea-pig polyclonal |
| PRLR | Epitope mapping within an extracellular domain of PRL-R of mouse origin | PRL-R Antibody (E-20): | sc-21816; Santa Cruz Biotechnology Inc., Germany | Goat polyclonal |
| TSH | Human βTSH (Chemicon International, Los Angeles, CA, USA) | Anti-hβTSH | G. Tramu (University of Bordeaux 1, Bordeaux, France) | Rabbit polyclonal |
| TSHR | Epitope mapping near the N-terminus of TSHR of human origin | TSHR Antibody (N-19) | sc-7816; Santa Cruz Biotechnology Inc., Germany | Goat polyclonal |
